# Role of Surfactant and pH in Dissolution of Curcumin

**DOI:** 10.4103/0250-474X.54280

**Published:** 2009

**Authors:** S. M. H. Rahman, T. C. Telny, T. K. Ravi, S. Kuppusamy

**Affiliations:** Department of Pharmaceutics, PSG College of Pharmacy, Peelamedu, Coimbatore-641 004, India; 1 Department of Pharmaceutical Analysis, Pulla Reddy Institute of Pharmacy, Jinnaram (M), Medak-502 313, India; 2 Department of Pharmaceutics, Sri Ramakrishna Institute of Paramedical Sciences, Sarojini Noidu Road, Coimbatore-641 044, India

**Keywords:** Curcumin, dissolution medium, sodium lauryl sulphate

## Abstract

Curcumin is a phytoconstituent with wide range of biological activity. It is poorly soluble in water. In the present study a new dissolution medium was developed, as there is no validated dissolution method available in the literature. The composition of the dissolution medium was selected on the basis of solubility data at 37°. Solubility data revealed that addition of surfactant may be suitable as a dissolution medium. The suitability of dissolution medium (0.5% sodium lauryl sulphate in water) relative to the other dissolution medium was evaluated. The selected dissolution media was used for the evaluation of curcumin tablets.

Curcumin is a potent phytoconstituent with wide range of biological activity[1]. Developing dissolution method for poorly soluble drugs has been a consistent challenge for the pharmaceutical scientists. The testing of pharmaceutical dosage forms for *in vitro* drug release and dissolution characteristics is very important for ensuring batch to batch quality control and to optimize formulations during drug development. Drugs, those are practically insoluble in water (less than 0.01%) need special attention during dissolution testing for proper *in vitro-in vivo* correlation. Since the dissolution may be a rate limiting step in the *in vivo* absorption process, there is a need for developing an appropriate dissolution medium[2].

Approaches usually used in the design of dissolution medium for poor water soluble drugs include increasing the volume of aqueous sink or removing the dissolved drug, solubilization of the drug by co-solvents (up to 40%) or by anionic or non-ionic surfactants (in post micellar concentration) or alteration of pH to enhance the solubility of insoluble drug molecules[3,4]. Among aforementioned approaches, pH modification and surfactant addition are the simplest and can be tailored to resemble GI fluid environment.

In the present investigation aqueous solubility of curcumin in medium containing co-solvents or surfactants was assessed to develop a dissolution system which satisfies sink condition for testing curcumin formulations. The selected dissolution media was used to study the dissolution process of curcumin tablets (300 mg).

Curcumin was a gift sample from M/s Natural Remedies Pvt. Ltd., Bangalore. Curcumin tablets were purchased from Shanjivani Phytopharma Pvt. Ltd., Mumbai. Sodium lauryl sulphate was purchased from S. D. Fine Chemicals, Mumbai. All other materials used were of analytical grade.

The apparent solubility of curcumin in water or in presence of co-solvents or surfactant in water was determined at 37°. Curcumin (50 mg) was added to 50 ml of water in an iodine flask and kept in temperature controlled magnetic stirrer maintained at 37° for 24 h. After shaking, the flasks were kept in incubator at 37±0.5° for equilibration for 12 h. Then the solution was filtered by whatman filter paper and the clear filtrate was assayed spectrophotometrically at 430 nm against blank solution. Form the available literature[5] the adsorption to Filter II (Whatman Filter) was much lower and in most cases negligible.

Dissolution experiments were performed using USP standard dissolution apparatus Type II (M/s Eletrolab, Mumbai) at 37° at a paddle speed of 100 rpm. The dissolution medium was 900 ml of either water or a mixer of water and SLS solution, selected on the basis of solubility data obtained from the experiments using 0.25, 0.5, 1.0, 1.5 and 2% of SLS in water. These mediums were also used to test the dissolution of bulk powder (100 mg, particle >200 μM) of curcumin. 10 ML Samples were withdrawn at periodical interval and analyzed spectrophotometrically at 430 nm. The same volume of dissolution medium maintained at 37° was added to maintain constant volume and sink condition.

In this study, solubility data was used as the basis for the development of a dissolution medium for curcumin. Since curcumin is poorly soluble in water, solubility determination was carried out using 0.25, 0.5, 1.0, 1.5 and 2% of SLS in water, phosphate buffer (pH 7.4 and 8) and acetate buffer (pH 4). The apparent solubility of curcumin in different media is given in the (Table 1).

**TABLE 1:** APPARENT SOLUBILITY STUDIES

Medium	Concentration μg/ml (n=3)
Water	152±5.5
0.1 N HCl	132±3.2
0.25% w/v SLS	132±3.2
0.5% w/v SLS	234±4.3
0.75% w/v SLS	465±5.2
1% w/v SLS	695±6.7
1.5% w/v SLS	782±5.8
2% w/v SLS	982±4.8
pH 7.4	348±3.2
pH 8	482±5.7
pH 4	231±4.2

Apparent solubility of curcumin in various dissolution medium at 37° is given, and the concentration is expressed as micrograms per milliliters.

Buffer solutions were prepared according to Indian Pharmacopoeia monographs. Phosphate buffer 7.4 was prepared by dissolving 2.38 g of disodium hydrogen phosphate, 0.19 g of potassium dihydrogen phosphate and 8.0 g of sodium chloride in sufficient water to produce 1000 ml. Phosphate buffer pH 8.0 was prepared by mixing 50 ml of 0.2 M potassium dihydrogen phosphate with 46.8 ml of 0.2 M sodium hydroxide and sufficient water to produce 500 ml. Acetate buffer pH 4 was prepared by taking 2.86 ml of glacial acetic acid and 1 ml of 50% w/v solution of sodium hydroxide in 100 ml volumetric flask, water was added to make up the volume and mixed.

The results indicated that dissolution rate of curcumin increased with increase in SLS concentration in dissolution medium and maximum dissolution was found in water containing 2% w/v of SLS. Addition of surfactant to the dissolution medium improves the dissolution of pure drug by facilitating the drug release process at the solid/liquid interface and micelle solubilization in the bulk[6]. Factors to consider when evaluating surfactants are cost and concentration needed to improve the dissolution of poorly soluble drugs. The solubility of drug can be enhanced by ensuring that the surfactant concentration is at least above the critical micellar concentration (CMC). The CMC will depend upon the surfactant itself and ionic strength of media. The amount of surfactant needed depends on the CMC and the degree to which the compound partitions into the micelles.[7] It may be the reason for the fact that increase in concentration of SLS above 2% did not exhibit any increase in the amount of curcumin dissolved. The concentration of SLS commonly used in dissolution media ranges from 0.1-3%[8]. The CMC of the SLS was estimated to be 0.03% in the literature[9].

For most poorly water-soluble drugs, pH of the dissolution medium has less effect on dissolution, but surfactants added to the dissolution medium will increase drug solubility significantly. A colloid system, which contains surfactant micelles, will help maintain a poorly water-soluble drug solubilized in an aqueous medium. The dissolution of the drug can be adjusted by changing the concentration of the surfactant in the medium. Sink conditions can be achieved by using higher concentrations of the surfactant. Up to 3% surfactant media are often used in dissolution of poorly water-soluble drugs[10]. However, the human gastrointestinal (GI) track does not have such a high concentration of surfactant, therefore it is not a surprise to find out that the dissolution results obtained from media of high surfactant concentrations have poor correlation with bioavailability. It is easy to understand that a bio-relevant medium will need a similar surface activity as bio-fluids.

Studies on SLS solutions[11] indicated that the surface tension of SLS solutions decreased dramatically above the critical micellar concentration (0.023%) and it reached a minimum surface tension at 0.2% with no significant change at higher concentrations. This suggested that a bio-comparable surface activity can be achieved at low surfactant concentrations (0.2%). In our lab conditions the low surface tension for the SLS was found to be 0.3%. Therefore the typical range of 0-0.5% SLS used in dissolution media is well above the CMC of SLS. Hence, 0.5% SLS was selected as dissolution medium when compared to lower concentration.

The performance of the selected dissolution medium (900 ml of 0.5% w/v of SLS in water) was confirmed by conducting dissolution experiments of prepared and commercial formulations and the results are shown in (figs. 1 and 2). The release rate of curcumin from commercial formulations was found to be more than 85% within 45 min under test conditions. The results of the present study clearly indicate that 0.5% w/v of SLS in water as dissolution medium is suitable for routine *in vitro* dissolution testing of curcumin formulations.

**Fig. 1:**
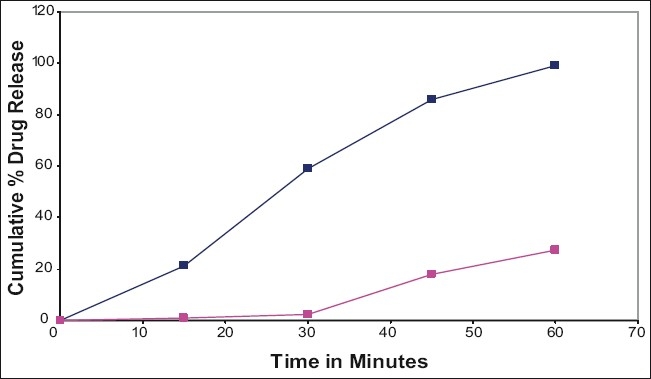
*In vitro* dissolution profile of commercial curcumin tablets. Comparison of *in vitro* dissolution profile of commercial curcumin tablets in water and 0.5% SLS

**Fig 2:**
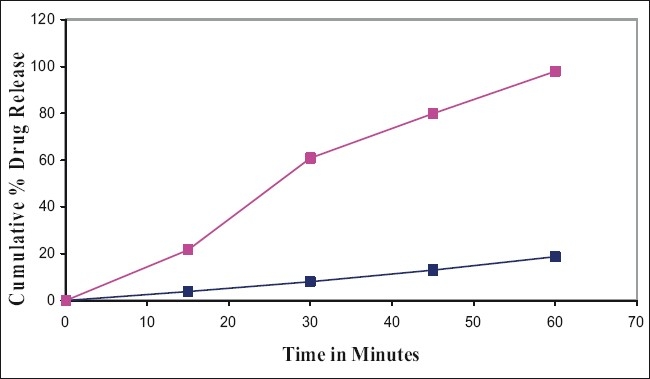
*In vitro* dissolution profile of prepared curcumin tablets. Comparison of *in vitro* dissolution profile of the prepared curcumin tablets in water and 0.5% SLS

## References

[1] Ishita C, Kausik B, Uday B, Ranajit KB (2004). Turmeric and Curcumin: Biological actions and medicinal applications. Current Sci.

[2] Gander B, Ventouras K, Gurny R, Doelker E (1985). *In vitro* dissolution medium with supramicellar surfactant concentration and its relevance for *in vivo* absorption. Int J Pharm.

[3] Gibaldi M, Feldman S (1967). Establishment of sink conditions in dissolution rate determinations: Theoretical consideration and application to nondisintegrating dosage forms. J Pharm Sci.

[4] Shah VP, Konecny JJ, Everett RL, McCullogh B, Noorizadeh AC, Skelly JP (1989). *In vitro* dissolution profile of water-insoluble drug dosage forms in the presence of surfactant. Pharm Res.

[5] Chiou WL, Smith DL (1970). Adsorption of organic compounds by commercial filter papers and its implication on quantitative-qualitative chemical analysis. J Pharm Sci.

[6] Abdou HM, Hanna S, Mohammad N, Gennaro AR (2000). interfacial phenomenon. Remington: The Science and Practice of Pharmacy.

[7] Schott H, Kwan LC, Feldman S (1982). The role of surfactant in the release of very slightly soluble drug from tablets. J Pharm Sci.

[8] Noory C, Tran N, Ouderkirk L, Shah V (2002). Steps for development of a dissolution test for sparingly water-soluble drug products. Am Pharm Rev.

[9] Zhao F, Malayev V, Rao VV, Hussain M (2004). Effect of Sodium Lauryl Sulfate in dissolution media on dissolution of hard gelatin capsule Shells. Pharm Res.

[10] Shah PV, Noory A, Noory C, McCullough B, Clarke S, Everett R (1995). *In Vitro* dissolution of sparingly water-soluble drug dosage forms. Int J Pharm.

[11] Sjokvist E, Nystrom C, Alden M, Caram-Lelham N (1992). Physicochemical aspects of drug release. XIV. The effects of some ionic and non-ionic surfactants on properties of a sparingly soluble drug in solid dispersions. Int J Pharm.

